# Acetabula Osteoid Osteoma Mimicking Juvenile Idiopathic Arthritis and Chronic Recurrent Multifocal Osteomyelitis

**DOI:** 10.1155/2020/8810735

**Published:** 2020-08-28

**Authors:** Hend Abd El Baky, Richard D. Thomas, Joseph Kuechle, Rabheh Abdul-Aziz

**Affiliations:** ^1^Department of Pediatric Rheumatology, University at Buffalo, Oishei Children's Hospital, Buffalo, NY, USA; ^2^Department of Pediatrics, Faculty of Medicine, Cairo University, Cairo, Egypt; ^3^Department of Radiology, University at Buffalo, Oishei Children's Hospital, Buffalo, NY, USA; ^4^Department of Orthopedics, Jacobs School of Medicine & Biomedical Sciences, Buffalo, NY, USA

## Abstract

Osteoid osteoma (OO) is a benign bone tumor that usually presents between 10 and 35 years of age. The metaphysis and diaphysis of the femur and tibia are the typical locations. The diagnosis is usually straightforward when images reveal a radiolucent nidus surrounded by reactive sclerosis. However, the diagnosis is more difficult when it occurs at atypical locations with nonspecific and misleading appearance on images. OO may mimic juvenile idiopathic arthritis (JIA), bone infection, or malignancy. We present a 14-year-old male with a 4-month history of left hip pain. His pain was worse with playing hockey and lacrosse and in the morning and sometimes woke him up at night. His examination was significant for pain with flexion and external rotation of the left hip and for mild limitation of full external rotation. Blood work revealed normal complete blood count, erythrocyte sedimentation rate, and C-reactive protein. Left hip X-ray was unremarkable. Left hip MR arthrogram showed marked edema of the medial and posterior walls of the left acetabulum. CT-guided biopsy of the left acetabulum showed unremarkable flow cytometry and chronic inflammatory component raising concern about chronic recurrent multifocal osteomyelitis (CRMO). Bone scan revealed focal increased uptake in the left acetabulum and no additional abnormality. Repeat MRI with intravenous contrast showed a left hip effusion, focal synovial enhancement in the medial left hip, and acetabula edema. The patient failed treatment for presumed JIA and CRMO with nonsteroidal anti-inflammatory drugs (NSAIDs), steroids, methotrexate, and adalimumab. CT scan of the left hip was performed for further evaluation of the bone and showed 11 × 6 mm low attenuation focus with subtle internal nidus in the posteromedial aspect of the acetabular rim, suggestive of intra-articular OO. Radiofrequency ablation was performed with no complications, and the left hip pain improved. The atypical location resulted in delay of diagnosis for 12 months after presentation. We highlight the diagnostic pitfalls observed in atypical OO locations and the difficulties this creates with making the diagnosis. OO mimicking JIA has previously been described. We submit CRMO as another differential diagnosis which may be mimicked and demonstrate the vital role of CT scan in the diagnosis.

## 1. Introduction

Osteoid osteoma (OO) is a benign tumor of bone, which was first described by Jaffe in 1935 [1]. It is the third most common benign bone tumor, accounting approximately for 13.5% of benign bone tumors and 3% of all primary bone tumors [2]. It typically affects children and adolescents between 10 and 35 years of age, and it affects males as twice as females [3]. The metaphysis and diaphysis of long bones, particularly the femur and tibia, are the typical locations for OO; however, it can occur anywhere [3]. Intra-articular OO (IAOO) is uncommon and accounts for 5–12% of all OOs [1], with the hip joint being the most common site [4]. The acetabulum is a rare site accounting approximately for 1% of all OOs [5]. Histologically, OO is composed of a central nidus composed of vascular osteoid tissue and woven bone lined by osteoblasts and surrounded by dense sclerotic bone [6].

The typical clinical presentation of OO is local pain that is most severe at night and can be relieved by nonsteroidal anti-inflammatory drugs (NSAIDs) [7]. Depending on the location of OO, patients may present with local swelling and tenderness, gait disturbance, bony deformity, or muscle atrophy. The diagnosis of OO can be confirmed by plain radiographs, technetium-99 m (Tc-99 m) bone scans, computed tomography (CT) scans, and magnetic resonance images (MRI). On plain radiographs, the tumor appears as a central radiolucent nidus surrounded by dense sclerotic mass. On CT scans, the lesion is usually a round or oval-shaped low-density nidus with a reactive peripheral sclerotic area [8]. MRI is used to detect soft tissue and bone marrow anomalies next to OO [8]. When OO occurs at atypical locations, they may have a nonspecific appearance on different imaging modalities, particularly on MRI [9].

OO can be managed conservatively. Invasive treatment is considered when pain is very severe and refractory to medications. Options include open or arthroscopic surgical excision of the tumor, CT-guided radiofrequency ablation, or CT-guided laser photocoagulation [10]. Treating cases of acetabular OO is challenging due to limited experience and difficult anatomical location being in close proximity to the sciatic nerve and triradiate cartilage [11].

OO is frequently misdiagnosed when it occurs in atypical locations as it may mimic juvenile idiopathic arthritis (JIA), bone infection, or malignancy [12]. Here, we described this rare case of acetabular OO. We aimed to address the diagnostic pitfalls and to highlight the difficulties associated with diagnosis.

## 2. Case Presentation

A 14-year-old male patient presented to our rheumatology clinic with a 4-month history of left hip pain. The pain was more severe in the morning and sometimes woke him up at night and also increased with practicing sports (hockey and lacrosse). His pain is 1-2 of 10 when he is resting and 7-8 of 10 when he is playing sports. There was no history of trauma. There was no associated swelling or morning stiffness, and no pain in any other joints.

**Figure 1:**
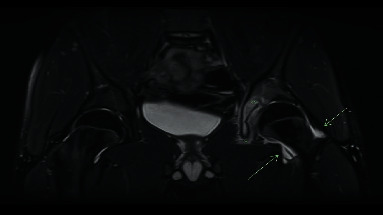
MRI T2 fat-saturated coronal image demonstrating acetabular and periacetabular soft tissue edema (*∗*) and a small joint effusion (arrow) in the left hip.

The patient was initially evaluated in the orthopedics clinic. Initial blood work revealed normal complete blood picture (CBC), erythrocyte sedimentation rate (ESR), and C-reactive protein (CRP). Left hip X-ray was normal. Initial MR arthrogram of the left hip showed marked edema of the medial and posterior walls of the left acetabulum (Figure 1). The patient was started on naproxen 220 mg (3.5 mg/kg) once daily, with limited improvement in terms of less frequent waking up at night from pain, knowing this is not optimal dose.

**Figure 2:**
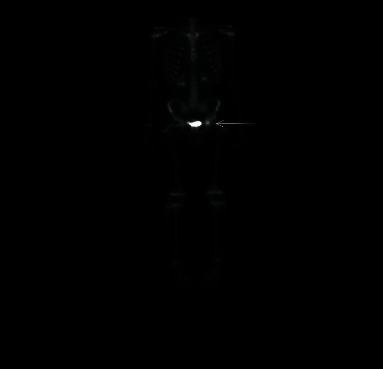
Bone scan demonstrating abnormal uptake in the left acetabulum, lateral to excreted isotope in the bladder (arrow).

With persistence of pain, CT-guided biopsy of the left acetabulum was performed and revealed unremarkable flow cytometry, and histopathology showed cortical bone with rare foci of marrow elements and a mild chronic inflammatory component and no evidence of malignancy. The patient was referred to the rheumatology clinic for further evaluation. Initial examination showed pain with flexion and external rotation with mild limitation of full external rotation of the left hip. JIA and CRMO could not be ruled out given his clinical examination and the histopathology result. The patient was started on prednisone 50 mg (0.7 mg/kg) once daily (that was gradually tapered), and naproxen dose was increased to 375 mg twice daily. Two months later, left hip pain continued, and he started to experience pain of the right hip, right metacarpophalangeal (MCPs), and left clavicle. He was started on weekly methotrexate in addition for presumed diagnosis of JIA and/or CRMO. Bone scan was unremarkable apart from focal increased uptake in the left acetabulum (Figure 2).

**Figure 3:**
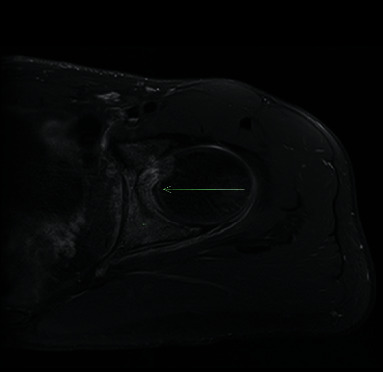
MRI contrast-enhanced T1 fat-saturated axial image demonstrating enhancing bone edema (^*∗*^) and medial synovial thickening with enhancement (arrow).

**Figure 4:**
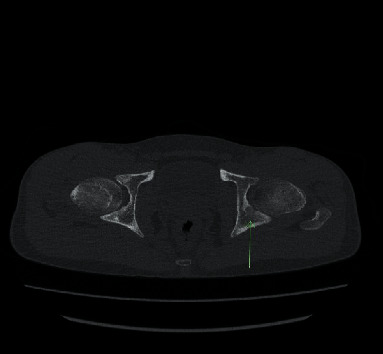
CT scan demonstrating a low attenuation intra-articular lesion with adjacent sclerosis (arrow).

After two months of methotrexate treatment, the patient reported improvement in the intensity and frequency of the left hip pain; however, he was still experiencing pain with certain positions. The pain of the right hip, left clavicle, and right MCPs resolved completely. Due to persistence of the left hip pain, adalimumab 40 mg subcutaneous every 2 weeks was added, with no significant improvement in pain. He reported increased need for NSAIDs (twice daily almost every day). Repeat contrast-enhanced MRI showed left hip effusion that decreased from before, with medial focal synovial enhancement and edema of the acetabulum (Figure 3). These findings are not typical for JIA where more diffuse synovial process would be anticipated. Pain was refractory to treatment for presumed JIA and CRMO with NSAIDs, steroids, methotrexate, and adalimumab. Subsequently, CT scan of the left hip was done for further evaluation. It showed 11 × 6 mm low attenuation focus with subtle internal nidus in the posteromedial aspect of the acetabular rim suggestive of intra-articular OO (Figure 4). Steroids, adalimumab, and methotrexate were discontinued. Uncomplicated radiofrequency ablation of the left acetabular OO was performed with complete resolution of the left hip pain.

## 3. Discussion

Over half of OO lesions occur in the lower extremity long bones, with the proximal femur being the most common location. The acetabulum is a rare site accounting for only 1% of all OOs [5]. Diagnosis of this rare site is challenging because of the nonspecific symptoms that may mimic other pathologies such as JIA, infections, malignancy, avascular necrosis, and traumatic conditions of the hip [12]. CRMO and JIA were initially considered in our patient, and he was treated accordingly. Intra-articular OO mimicking JIA has been previously described in few cases [12, 13]. To our knowledge, we are the first to report a case of acetabular OO with similar presentation to CRMO. This leads to a delay of 16 months before the correct diagnosis was reached. Previous studies reported the mean delay between the onset of symptoms and the diagnosis of intra-articular OO from 2 months to 10 years [14]. Szendroi et al. reported in a small series that the duration of symptoms before the diagnosis (27 months) for intra-articular or juxta-articular locations is 3 times longer than for extra-articular sites [4, 15]. This delay in diagnosis exposed the patient to unnecessary medications with potential side effects as well as delay of the appropriate treatment which could result in joint damage for intra- or juxta-articular cases of OO, highlighting the importance of prompt detection and adequate early treatment [16].

Despite the advent improvement in imaging, diagnosis of atypical location of OO remains a challenge. Radiographic features of hip IAOO may differ from extra-articular lesions. Unlike extra-articular locations, plain radiographs provide only subtle findings due to the absence of perilesional sclerosis or periosteal reaction [17]. On MRI, in 35%, the nidus cannot be detected because of significant edema that surrounds the tumor, while the nidus has atypical morphology in 50% of the cases, leading to misdiagnosis [18]. While MRI is often the imaging modality of choice for assessment of hip and groin pain, the diagnostic accuracy of MRI for detection of atypical OO is lower than that of CT [18]. In this case study, MRI could not provide early diagnosis of OO, and the lesion was detected by CT scan. Bone scintigraphy is highly sensitive but demonstrates lower specificity compared to CT scan, especially in intra-articular locations as bone sclerosis around the nidus cannot be detected because of decreased uptake associated with synovial reaction [19]. CT is the gold standard for diagnosis as it is the most useful imaging modality to identify the OO nidus which appears as central calcification and surrounding sclerosis [4].

Surgical treatment for acetabular OO is challenging because of limited surgeon experience with therapeutic procedures and the complex anatomical location with proximity to the sciatic nerve and triradiate cartilage. Although numerous surgical approaches to acetabular OO have been described, including open surgery, arthroscopy, and CT-guided approaches, optimal management for acetabular OO has not been established [5]. CT-guided radiofrequency ablation is currently the treatment of choice for pediatric patients as it is safe, effective, and less invasive [20]. Our patient was treated with CT-guided radiofrequency ablation with no complications reported and with complete resolution of pain.

## 4. Conclusion

OOs in atypical locations are difficult to diagnose due to nonspecific presentation and misleading appearance on imaging (MRI and bone scan). Thorough history and clinical examination together with CT scan are vital for diagnosis.
